# Self-Reported Sitting Time in New York City Adults, The Physical Activity and Transit Survey, 2010–2011

**DOI:** 10.5888/pcd12.140488

**Published:** 2015-05-28

**Authors:** Stella S. Yi, Katherine F. Bartley, Melanie J. Firestone, Karen K. Lee, Donna L. Eisenhower

**Affiliations:** Author Affiliations: Katherine F. Bartley, Melanie J. Firestone, Karen K. Lee, Donna L. Eisenhower, New York City Department of Health and Mental Hygiene, New York, New York.

## Abstract

**Introduction:**

Recent studies have demonstrated the negative health consequences associated with extended sitting time, including metabolic disturbances and decreased life expectancy. The objectives of this study were to characterize sitting time in an urban adult population and assess the validity of a 2-question method of self-reported sitting time.

**Methods:**

The New York City Health Department conducted the 2010–2011 Physical Activity and Transit Survey (N = 3,597); a subset of participants wore accelerometers for 1 week (n = 667). Self-reported sitting time was assessed from 2 questions on time spent sitting (daytime and evening hours). Sedentary time was defined as accelerometer minutes with less than 100 counts on valid days. Descriptive statistics were used to estimate the prevalence of sitting time by demographic characteristics. Validity of sitting time with accelerometer-measured sedentary time was assessed using Spearman’s correlation and Bland-Altman techniques. All data were weighted to be representative of the New York City adult population based on the 2006–2008 American Community Survey.

**Results:**

Mean daily self-reported sitting time was 423 minutes; mean accelerometer-measured sedentary time was 490 minutes per day (*r* = 0.32, *P* < .001). The mean difference was 49 minutes per day (limits of agreement: −441 to 343). Sitting time was higher in respondents at lower poverty and higher education levels and lower in Hispanics and people who were foreign-born.

**Conclusion:**

Participants of higher socioeconomic status, who are not typically the focus of health disparities–related research, had the highest sitting times; Hispanics had the lowest levels. Sitting time may be accurately assessed by self-report with the 2-question method for population surveillance but may be limited in accurately characterizing individual-level behavior.

## MEDSCAPE CME

Medscape, LLC is pleased to provide online continuing medical education (CME) for this journal article, allowing clinicians the opportunity to earn CME credit.

This activity has been planned and implemented in accordance with the Essential Areas and policies of the Accreditation Council for Continuing Medical Education through the joint sponsorship of Medscape, LLC and *Preventing Chronic Disease*. Medscape, LLC is accredited by the ACCME to provide continuing medical education for physicians.

Medscape, LLC designates this Journal-based CME activity for a maximum of 1 ***AMA PRA Category 1 Credit(s)™***. Physicians should claim only the credit commensurate with the extent of their participation in the activity.

All other clinicians completing this activity will be issued a certificate of participation. To participate in this journal CME activity: (1) review the learning objectives and author disclosures; (2) study the education content; (3) take the post-test with a 75% minimum passing score and complete the evaluation at www.medscape.org/journal/pcd; (4) view/print certificate.

**Release date: May 28, 2015; Expiration date: May 28, 2016**

### Learning Objectives

Upon completion of this activity, participants will be able to:

- Evaluate the potential health impact of sitting time
- Distinguish mean durations of daily sitting time in the current study
- Assess variables associated with higher mean durations of self-reported sitting time in the current study
- Assess variables associated with higher mean durations of accelerometer-reported sitting time in the current study

**EDITORS**

Camille Martin, Editor, *Preventing Chronic Disease*. Disclosure: Camille Martin has disclosed no relevant financial relationships.

**CME AUTHOR**

Charles P. Vega, MD, Clinical Professor of Family Medicine, University of California, Irvine

Disclosure: Charles P. Vega, MD, has disclosed the following relevant financial relationships: Served as an advisor or consultant for: Lundbeck, Inc; McNeil Pharmaceuticals; Takeda Pharmaceuticals North America, Inc.

**AUTHORS AND CREDENTIALS**

Stella S. Yi, PhD, MPH, Department of Population Health, New York University School of Medicine, New York, New York. Katherine F. Bartley, Melanie J. Firestone, Karen K. Lee, Donna L. Eisenhower, New York City Department of Health and Mental Hygiene, New York, New York.

Disclosures: Stella S. Yi, PhD, MPH; Katherine F. Bartley, PhD; Melanie J. Firestone, MPH; Karen K. Lee, MD; Donna L. Eisenhower, PhD have disclosed no relevant financial relationships.

## Introduction

Sitting is a sedentary behavior and has been linked to all-cause and cardiovascular disease mortality and to decreased life expectancy; 3 hours per day of sitting leads to a life expectancy decrease of 2 years (1–3). Sitting is distinct from a lack of recreational or nonrecreational physical activity (PA), and these behaviors have differing demographic determinants (4). Furthermore, these distinct behaviors may affect health outcomes via alternative biological mechanisms (5). This research has given rise to the “inactivity physiology paradigm,” or that “sitting too much is not the same as lack of exercise, and as such, has its own unique metabolic consequences” such as decreased lipoprotein lipase activity in skeletal muscles in the legs (5). People who exercise regularly but are still sedentary for several hours a day may have a risk of adverse health outcomes that is higher than would be expected given their overall PA levels, although recent findings in the literature are mixed (6).

A common practice is to use a broad definition of *sedentary*, although specific definitions are needed (7,8); this investigation thus focused on sitting time. Measured estimates in New York City (NYC) have not been previously published and are of interest, given the differences in PA levels between NYC and US adults (9). In a report released by the NYC Health Department, adult New Yorkers were 3 times as likely as adults nationwide to meet PA recommendations (29% versus 11%) (9). Previous surveys have also assessed sitting time by using 1 or 2 questions on weekdays versus weekends (10) or up to 21 questions to capture specific domains of sitting time (11). However, these prior question sets may not adequately address sitting time in a single day or may be too long to administer at the population level. Recall would likely be improved if the single day were divided into smaller periods. 

We used 2 questions derived from 1 previously standardized question, dividing a single day into daytime and evening sitting times to improve recall and estimation. The objectives of this analysis were to describe self-reported sitting time and accelerometer-measured sedentary time in a diverse, urban population and to test the validity of a 2-question method of self-reported sitting time that allowed for discrete periods of recall. 

## Methods

The NYC Health Department conducted the Physical Activity and Transit (PAT) Survey, a cross-sectional, assessment of PA and transit behaviors in NYC adults. The PAT Survey was a random-digit–dial telephone survey designed to provide estimates of PA at the city, borough (county), and subgroup (eg, race/ethnicity) levels. An overlapping landline and cellular telephone sample frame to contact adults in residential households in NYC — with disproportionate, equal-sized samples from the 5 boroughs — was used, and areas with higher levels of obesity were oversampled. The first wave of interviews was conducted from September through November 2010 (n = 1,323); the second wave was conducted from March through November 2011 (n = 2,488; N = 3,811). The PAT Survey and device portion have been described previously (9,12,13). The institutional review board of the NYC Health Department approved this study as human subjects research.

### Measurement and definitions of covariates

Self-reported sitting time was assessed by combining responses from 2 questions on time spent sitting for daytime and evening: “On an average day during the last 7 days, from the time you woke up to around 5 o’clock in the evening, how many hours or minutes did you spend sitting?” and “From 5 o’clock in the evening to the time you went to bed on an average day during the last 7 days, how many hours or minutes did you spend sitting?” Demographic characteristics (age, sex, race/ethnicity, poverty status, education, nativity) and height and weight were self-reported. Race/ethnicity was assessed using 2 questions on Hispanic ancestry and race group and was categorized as non-Hispanic white, non-Hispanic black, Hispanic, non-Hispanic Asian, and non-Hispanic other. Poverty level was based on annual combined household income and was grouped according to federal poverty guidelines (<200%, 200%–399%, ≥400% of the federal poverty level [FPL]). Nativity was defined as self-reporting being born in the United States or elsewhere. Puerto Ricans and respondents born in other US territories were considered US-born. Body mass index (BMI) was calculated from self-reported height and weight (kg/m^2^), and participants were categorized as underweight/normal (BMI <25.0), overweight (BMI of 25.0–29.9), or obese (BMI ≥30.0).

### Accelerometer subset

Mobile individuals (people who could walk >10 feet) in the second wave of the PAT Survey were asked to participate in a device follow-up study using accelerometers. Participants were asked to wear the accelerometer for 1 week during all waking hours and to remove it only when in water. Of those who completed an interview in the second wave of the PAT Survey, 803 (32%) agreed to participate and returned devices with data. Participants wore hip-mounted ActiGraph GT3X accelerometers (ActiGraph, LLC). Because they wore the devices at home and while working, while commuting, and during recreational time, data are comparable to the self-report data. To process the accelerometer data for analysis, activity thresholds from the National Health and Nutrition Examination Survey (NHANES) were used to assign minutes as sedentary time. Sedentary time was defined as accelerometer minutes with fewer than 100 counts on valid days (≥10 hours of wear time).

### Statistical methods

All analyses of self-reported and accelerometer data were restricted to mobile participants, those with plausible PA values, and responses to the questions on sitting time; the final analytic sample size for the self-report data was 3,597 (94.4% of 3,811 participants). The minimum accelerometer wear time for a reliable estimate of weekly activity is 10 or more hours on 4 or more days (14). With this cutoff as the inclusion criterion, 667 participants (83.1% of 803 accelerometer participants) were included in the final analytic data set for accelerometer-related analyses.

Validity of self-reported sitting time with accelerometer-measured sedentary time was assessed by using Spearman’s correlation and agreement methods outlined by Bland and Altman (15). Linear regression was used to check whether the mean difference and limits of agreement (LoA, ±2 standard deviations) varied across average values of self-reported sitting time and accelerometer-measured sedentary time ([self-reported sitting time + accelerometer sedentary time]/2).

Mean self-reported sitting time and accelerometer-measured sedentary time was assessed overall and stratified by demographic characteristics; differences were assessed using *t* tests. Self-report of day and evening sitting time was also stratified by demographics, and differences were assessed using *t* tests. Pearson correlations of sitting time to sedentary time were also run separately for day and for evening sitting. Multivariable linear regression models adjusted for age, sex, race/ethnicity, poverty status, education level, nativity, and BMI category were used to assess the factors associated with sitting time. To estimate results representative of the NYC population, analyses incorporated sampling weights to account for complex survey design and nonresponse. Data were analyzed using SUDAAN (version 10.0; Research Triangle Institute) and SAS (version 9.2, Research Triangle Institute).

## Results

Seventy percent of respondents were aged 25 to 64 years, more than half were female, nearly half were black or Hispanic, and almost 40% had an income of less than 200% of the FPL and were foreign-born (Table 1). The subset of participants who wore the accelerometer was similar to the overall sample, although they were slightly more likely to be US-born versus foreign-born.

**Table 1:** Demographic Characteristics of Participants, Physical Activity Transit Survey, 2010–2011

Characteristic	All Participants, Self-Report		Subset Participating in Accelerometer Component	
	N (%)	Weighted^a^ n (Weighted %)	N (%)	Weighted n (Weighted %)
**Total sample**	3,597 (100)	6,196,000 (100)	667 (100)	5,980,000 (100)
**Age group, y**				
18–24	262 (7.0)	760,000 (13.4)	40 (5.9)	759,000 (12.9)
25–44	1,132 (31.5)	2,375,000 (41.8)	217 (32.6)	2,479,000 (42.0)
45–64	1,383 (38.5)	1,762,000 (31.0)	291 (41.9)	1,838,000 (31.1)
≥65	812 (22.6)	789,000 (13.9)	128 (19.6)	832,000 (14.1)
**Sex**				
Male	1,470 (40.9)	2,692,000 (47.3)	263 (39.3)	2,782,000 (47.1)
Female	2,127 (59.1)	3,000,000 (52.7)	404 (60.7)	3,127,000 (52.9)
**Race/ethnicity**				
Non-Hispanic white	1,578 (43.9)	2,083,000 (36.6)	293 (44.2)	2,120,000 (35.9)
Non-Hispanic black	851 (23.7)	1,241,000 (21.8)	179 (26.8)	1,304,000 (22.1)
Hispanic	808 (22.5)	1,509,000 (26.5)	150 (22.4)	1,524,000 (26.1)
Non-Hispanic Asian	285 (7.9)	739,000 (13.0)	32 (4.7)	753,000 (12.7)
Other	75 (2.1)	121,000 (2.1)	13 (1.9)	191,000 (3.2^b^)
**Poverty/income^c^, % of federal poverty level**				
<200	1,288 (38.0)	2,328,000 (43.4)	220 (34.5)	2,213,000 (38.7)
200–399	585 (17.3)	853,000 (15.9)	122 (18.8)	1,135,000 (19.8)
≥400	1,315 (38.8)	1,698,000 (31.6)	275 (42.3)	1,841,000 (32.2)
**Education**				
Less than high school	445 (12.4)	1,057,000 (18.6)	56 (8.6)	1,147,000 (19.4)
High school	893 (24.9)	1,403,000 (24.7)	153 (22.9)	1,473,000 (24.9)
Some college	772 (21.5)	1,339,000 (23.6)	154 (23.2)	1,346,000 (22.8)
College graduate	1,479 (41.2)	1,880,000 (33.1)	304 (45.4)	1,943,000 (32.9)
**Nativity^d^**				
US born	2,232 (62.1)	2,999,000 (52.7)	463 (68.9)	3,242,000 (54.9)
Foreign born	1,362 (37.9)	2,687,000 (47.3)	204 (31.1)	2,667,000 (45.1)
**Body mass index category (kg/m^2^)**				
Underweight/normal (<25.0)	1,398 (39.1)	2,470,000 (43.6)	244 (37.1)	2,499,000 (42.8)
Overweight (25.0–29.9)	1,221 (34.1)	1,731,000 (30.6)	231 (24.8)	1,868,000 (32.0)
Obese (≥30.0)	959 (26.8)	1,461,000 (25.8)	189 (28.1)	1,476,000 (25.3)

a Data weighted to be representative of the New York City adult population based on the 2006–2008 American Community Survey.

b Estimate’s relative standard error (a measure of estimate precision) is greater than 30% or the sample size is less than 50, making the estimate potentially unreliable.

c “Don’t know” category is not presented, so percentages do not sum to 100%.

d Nativity was defined as self-reporting being born in the United States or elsewhere. Puerto Ricans and respondents born in other US territories were defined as being US-born.

### Sitting and sedentary time in NYC adults

**Figure 1:**
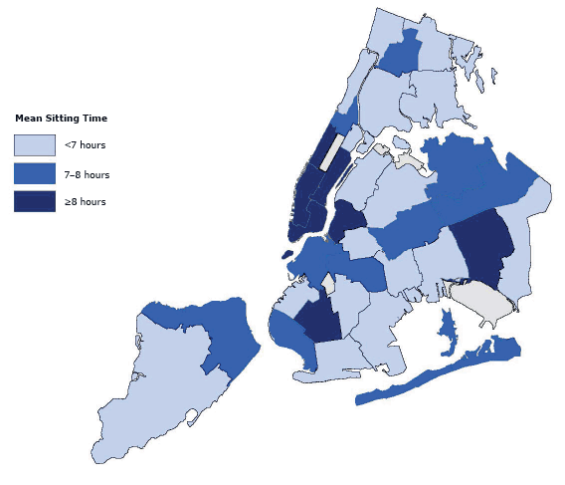
Mean Sitting Time, by United Hospital Fund, 34 Neighborhoods in New York City, Physical Activity and Transit Survey, 2010–2011. Darker areas indicate longer sitting times for neighborhood residents. **Table:** 

Self-reported mean daily sitting time was 423 minutes per day (7.1 hours/d), and accelerometer-measured mean daily sedentary time was 490 minutes per day (8.2 hours/d) (Table 2). High poverty individuals or those with lower education reported less sitting time per day. Self-reported sitting time was lower in Hispanics (324 min/d vs 465 min/d in non-Hispanic whites); it was also lower in women and those who were foreign-born and obese. Mean self-reported sitting time during daytime hours was 243 minutes per day (4.0 hours/d); during evening hours it was 180 minutes per day (3.0 hours/d) (Appendix). Mean sitting time was 8 or more hours in residents in Manhattan (Upper West Side, Upper East Side–Gramercy, Chelsea–Village, Union Square, Lower Manhattan) and in 2 areas of Brooklyn (Borough Park, Greenpoint) and Jamaica, Queens (Figure 1).

**Table 2:** Mean Self-Report of Sitting Times and Accelerometer-Measured Sedentary Times, by Demographic Characteristics, Physical Activity Transit Survey, 2010–2011

Characteristic	Self-Reported Sitting Time		Accelerometer-Measured Sedentary Time	
	Mean min/d (95% CI)	*P* Value^a^	Mean min/d (95% CI)	*P* Value^a^
**Overall**	423 (411–434)	NA	490 (474–506)	NA
**Age group, y**				
18–24	405 (374–437)	.14	449^b^ (382–517)	.40
25–44	434 (414–454)	1 [Reference]	480 (458–503)	1 [Reference]
45–64	417 (398–436)	.23	501 (477–524)	.21
≥65	418 (394–443)	.33	532 (511–554)	<.001
**Sex**				
Male	440 (422–458)	1 [Reference]	490 (462–517)	1 [Reference]
Female	407 (392–422)	<.001	490 (473–508)	.96
**Race/ethnicity**				
Non-Hispanic white	465 (450–480)	1 [Reference]	513 (498–528)	1 [Reference]
Non-Hispanic black	441 (416–466)	.11	494 (471–517)	.18
Hispanic	324 (302–345)	<.001	428 (396–460)	<.001
Non-Hispanic Asian	471 (434–509)	.75	550^b^ (495–606)	.20
Other	448 (375–521)	.66	475^b^ (448–502)	.02
**Poverty/income, % federal poverty level**				
<200	375 (356–394)	<.001	493 (469–516)	.33
200–399	421 (394–448)	<.001	504 (470–538)	.85
≥400	492 (473–511)	1 [Reference]	508 (487–528)	1 [Reference]
**Education**				
Less than high school	328 (294–361)	.01	430 (390–469)	<.001
High school	383 (363–403)	<.001	500 (474–525)	.03
Some college	444 (424–464)	<.001	467 (435–498)	<.001
College graduate	491 (473–509)	1 [Reference]	535 (517–553)	1 [Reference]
**Nativity^c^**				
US born	454 (440–468)	1 [Reference]	497 (480–515)	1 [Reference]
Foreign born	388 (369–406)	<.001	481 (453–508)	.32
**Body mass index category (kg/m^2^)**				
Underweight/normal (<25.0)	437 (418–456)	1 [Reference]	510 (487–532)	1 [Reference]
Overweight (25.0–29.9)	421 (402–440)	.25	466 (436–496)	.02
Obese (≥30.0)	404 (383–425)	.02	491 (462–519)	.30

Abbreviations: CI, confidence interval; NA, not applicable.

a *P* values determined using *t *tests for proportions.

b Estimate’s relative standard error (a measure of estimate precision) is greater than 30% or the sample size is less than 50, making the estimate potentially unreliable.

c Nativity was defined self-reporting being born in the United States or elsewhere. Puerto Ricans and respondents in other US territories were defined as being US-born.

United Hospital Fund Code	Mean Sitting Time, Hours
101	<7
102	<7
103	7–8
104	<7
105, 106, 107	<7
201	≥8
202	7–8
203	7–8
204	<7
205	<7
206	≥8
207	<7
208	<7
209	7–8
210	<7
211	<7
301	<7
302	7–8
303	<7
304	≥8
305, 307	≥8
306, 308	≥8
309, 310	≥8
401	<7
402	<7
403	7–8
404, 406	7–8
405	7–8
407	<7
408	≥8
409	<7
410	7–8
501, 502	7–8
503, 504	<7

Results using accelerometer data differed slightly from self-reported data; among participants aged 65 or older, measured sedentary time (532 min/d) was higher than self-reported sedentary time (418 min/d). Accelerometer data were similar in men and women (both 490 min/d). Statistical relationships with education level remained consistent across the 2 measures, though the dose–response effect across categories was not as apparent in the accelerometer data. No meaningful differences were observed by poverty level, nativity, or obesity in accelerometer data.

In multivariable models after adjustment for covariates (age, sex, race/ethnicity, poverty status, education level, nativity, and BMI category), self-reported sitting time was significantly higher in men than in women, highest in Asians and lowest in Hispanics, lower in those at lower education levels than in those at higher education levels, lower in those at higher poverty levels than in those at lower poverty levels, and lower in those who were foreign-born than in those who were US-born (Table 3). Accelerometer-assessed sedentary time was higher in older adults than in younger adults, lower in Hispanics than in other racial/ethnic groups, and lower among those with lower education levels than among college graduates.

**Table 3:** Beta Coefficients, Differences Associated with Self-Reported Sitting Time and Accelerometer-Measured Sedentary Time, Physical Activity Transit Survey, 2010–2011

Characteristic	Self-Reported % Sitting Time		Accelerometer-Measured % Sedentary Time	
	β, min/d (95% CI)	*P* Value^a^	β, min/d (95% CI)	*P* Value^a^
**Age group, y**				
18–24	−3.3 (−42.3 to 35.6)	.87	−0.21 (−49.3 to 48.9)	.95
25–44	1 [Reference]			
45–64	−8.0 (−33.3 to 17.4)	.54	17.2 (−7.1 to 41.6)	.17
≥65	−3.6 (−36.1 to 28.9)	.83	60.4 (35.5 to 85.3)	<.001
**Sex**				
Male			1 [Reference]	
Female	−29.51 (−7.0 to −52.1)	.01	−3.5 (−27 to 19.9)	.77
**Race/ethnicity**				
Non-Hispanic white	1 [Reference]			
Non-Hispanic black	20.6 (−11.5 to 52.7)	.21	−7.7 (−34.9 to 19.5)	.58
Hispanic	−66.3 (−96.1 to −36.5)	<.001	−42.3 (−71.2 to −13.4)	<.001
Non-Hispanic Asian	57.7 (14.6 to 100.7)	.01	42.7 (−6.2 to 91.5)	.09
Other	10.3 (−63.5 to 84.2)	.78	−13.0 (−47 to 21)	.45
**Poverty/income, % federal poverty level**				
<200	−40.0 (−70.7 to −9.2)	.01	40.9 (11.7 to 70.1)	.01
200–399	−36.4 (−69.3 to −3.5)	.03	15.1 (−17.7 to 48)	.37
≥400	1 [Reference]			
**Education**				
Less than high school	−94.9 (−136.7 to −53.2)	<.001	−89.1(−129.5 to −48.7)	<.001
High school	−77.9 (−107.1 to −48.7)	<.001	−37.8 (−65.8 to −9.7)	.01
Some college	−22.3 (−52.1 to 7.5)	.14	−53.1 (−90.6 to −15.7)	.01
College graduate	1 [Reference]			
**Nativity^b^**				
US born	1 [Reference]			
Foreign born	−43.7 (−68.1 to −19.2)	<.001	−7.0 (−30.1 to 16.1)	.55
**BMI category (kg/m^2^)**				
Underweight/normal (<25.0)	1 [Reference]			
Overweight (25.0–29.9)	−5.7 (−32.8 to 21.4)	.68	−14.2 (−40.9 to 12.5)	.30
Obese (≥30.0)	1.4 (−27.5 to 30.3)	.93	2.3 (−22.1 to 26.6)	.86

a *P* values determined using linear regression analyses.

b Nativity was defined as self-reporting being born in the United States or elsewhere. Puerto Ricans and respondents born in other US territories were defined as being US-born.

### Assessing validity of self-reported sitting

**Figure 2:**
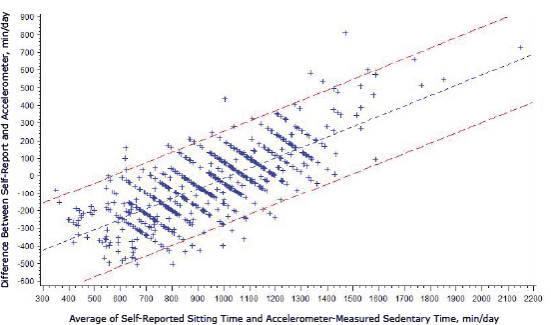
Bland-Altman plot for self-reported sitting time and accelerometer-measured sedentary time (min/d), Physical Activity Transit Survey, 2010–2011 (N = 667).

The correlation between self-reported sitting time and accelerometer-assessed sedentary time was modest (*r *= 0.32, *P* < .001); the correlation was stronger in daytime (*r* = 0.37, *P* < .001) versus evening (*r* = 0.23, *P* < .001) sitting. Correlations were highest among younger adults (aged 18–24 y; *r* = 0.46, *P* < .001) and those with higher incomes (≥400% FPL; *r* = 0.42, *P* < .001). Correlations were lowest for black (*r* = 0.24, *P* < .001), Hispanic (*r* = 0.27, *P* < .001), low-income (<200% FPL; *r* = 0.19, *P* < .001), foreign-born (*r* = 0.28, *P* < .001), and obese (*r* = 0.17, *P* = .02) participants. The Bland-Altman plot shows a scatterplot of the difference between measures by the average of the 2 measures (Figure 2). The mean difference between self-report and measurement was 49 minutes per day. The LoA were −441 to 343 minutes per day, meaning that an estimate of sitting time could be underreported by as much as 7.4 hours per day or overreported by 5.7 hours per day. Linear regression showed a significant positive association for the difference between self-reported sitting time and accelerometer-measured sedentary time. At lower levels of sitting time, self-report was lower than accelerometer-measured sedentary time. At higher levels of sitting time, self-report was higher than accelerometer-measured sedentary time (β = 0.59; standard error = 0.02; *P* < .001; LoA = mean difference ± 200.34).

## Discussion

In a representative sample of noninstitutionalized NYC adults, mean self-reported sitting time was 423 minutes per day (7.1 hours/d). Accelerometer-assessed sedentary time was 490 minutes per day (8.2 hours/d). These values are similar to national values of sedentary time assessed using accelerometer data from NHANES 2003–2004, which showed that the average US adult was sedentary for 7.7 hours per day (16). Mean sitting and sedentary time was highest in adults 65 years or older and in those who were college educated; accelerometer measurements demonstrated that these groups were sedentary for 8.9 hours per day.

Hispanics had the lowest values for self-reported sitting and measured sedentary time of all racial/ethnic groups. These results may reflect more physical occupations among Hispanics (17) but also point to the value of incorporating questions on both leisure-time and occupational activity when assessing PA levels in Hispanic populations. Similar results on occupation-related PA levels in Mexican Americans have been demonstrated in national data (18).

Although variations were seen in sitting times (lower values for Hispanics and those with lower educational levels and incomes), the amount of sitting time across all sociodemographic groups greatly exceeded the 3 hours per day associated with decreased life expectancy. Interventions for decreasing sitting time are needed to improve health outcomes across all groups. Worksite and home interventions should target all sociodemographic groups; however, the interventions should consider sex, age, and cultural and socioeconomic differences, as well as the time of day the sitting is occurring.

The main challenge of research on sedentary behavior is in defining it (7,8). This analysis focused on sitting time as a distinct sedentary behavior. We found that a 2-question method to assess sitting time demonstrated modest correlation with measurements of sedentary time by accelerometer (*r* = 0.32, *P* < .001), which is low compared with correlations of other self-reported and objective measures but similar to those observed in the PA literature (19). In a sample of Belgian adults aged 65 or older (n = 508), similar results were reported for validation of self-reported sitting time using a 21-question instrument and accelerometer-assessed sedentary time (11). The authors reported, in comparing self-report to measured values, a correlation of *r* = 0.30, a mean difference of 82 minutes per day, and wide LoA (−364 to 200 min/d) (11). The modest correlations in our and prior research may be because sitting is a subset of sedentary time, which includes a broader range of behaviors, such as reclining and lying down. This is reflected in the correlation data where the daytime correlation (*r *= 0.37) was larger than that observed for evening (*r* = 0.23) between self-report and accelerometer data.

The overall mean difference between self-report and accelerometer was 49 minutes per day, and wide LoA was observed in the Bland-Altman plot (−441 to 343 min/d). For population surveillance, cost and ease of administration are usually prioritized over more intensive and accurate measures, and questionnaires are often the only feasible method for assessing PA (20). Despite limitations to questionnaires for assessing PA accurately, they are useful in characterizing groups, providing rankings among subgroups, or both (21). Results from the plot corroborate this evidence and reinforce the notion that sitting time may be accurately assessed by self-report using the 2-question method for population surveillance but may be limited in accurately characterizing individual-level behavior.

NYC is among the most walkable cities in the nation. Recent analysis using an NYC-specific walkability scale that incorporated 5 equally-weighted components (residential density, intersection density, land use, subway stop density, and ratio of retail building floor area to retail land area) quantitatively illustrated the walkability of NYC; Manhattan in particular, was flagged as an area with high walkability (22). The geographic location of increased sitting time (ie, concentration in Manhattan) observed in this study is noteworthy and points to opportunities for intervention design. For example, given the high sitting times and favorable walking conditions in Manhattan, interventions that introduce breaks in sitting time with short walks are feasible. This finding highlights the potential for intervention through locating worksites in walkable neighborhoods in the United States and considering walkability in the design of new business districts. Similar intervention strategies could be implemented in other urban populations with comparable geographic concentrations of these characteristics.

In addition to promoting walking breaks among workers in walkable neighborhoods, other workplace interventions are being considered in NYC and elsewhere. A Cochrane review concluded that using a sit–stand desk with or without additional interventions such as information or counseling reduced sitting time by 113 minutes per workday; however, the quality of evidence was low, because the available studies had small numbers of participants and low-quality research design (23). Complementary to interventions in the workplace or during the day, strategies that target sitting time in the evening, such as encouraging movement during television watching, should be considered.

In NYC, promotion of stair use is also being used to provide opportunities for small bouts of physical activity throughout the day. The NYC Health Department has worked with the Department of Citywide Administrative Services to open stairwells in many publicly owned worksite buildings, coupled with posting of stair prompt signage encouraging people to “Burn Calories, Not Electricity. Take the Stairs!” Use of this signage is associated with stair use among occupants of multiple NYC buildings (24,25).

One of the main strengths of this analysis was the assessment of behaviors and exploration of validity of self-reported sitting time in an urban and diverse sample of adults. The availability of accelerometer data was an additional strength; however, the accelerometer is not able to distinguish between different domains of sedentary time (26,27) and may not be as accurate a method for validating self-report of sitting time as a device that distinguishes between sitting and lying down. However, given the absence of a “gold standard” for measuring sitting time, the accelerometer-assessed sedentary time may be considered as a fair proxy (28). Accelerometer technology has advanced to produce devices that distinguish between standing and lying down, but such devices may not always function properly. For instance, we set the model we used for this investigation to estimate standing versus lying down; however, we found that the setting did not work. Measurement of sedentary behavior is still a nascent field (29), and careful consideration should be given to the best required method for the specific sedentary behavior and question under study (27). Therefore, the focus on sitting time specifically was an additional strength of this study. Also, it may be easier to report and recall one specific sedentary behavior than multiple sedentary behaviors under one inclusive definition. 

This study had limitations. Validation of a 2-item versus 1-item question assessing sitting time would have been more appropriately assessed by comparison with each other; however, standards of practice for improving a single question from an established instrument were applied by using a previously validated single-item question and by breaking up the period of recall into 2 periods. Although data were weighted to be representative of the NYC adult population, the small sample sizes of some subgroups (eg, Asian) limited external validity of findings in these groups. Citywide results, however, may be generalizable to other urban areas with similar urban landscapes and opportunities for walking and active transport. Finally, to successfully quantify sitting time, it has been suggested to incorporate both measured data and context-specific information (28,30). We did not have this level of information available for this study, given that the PAT Survey was designed for citywide surveillance across multiple domains of PA and active transport.

The 2-question method to quantify sitting time is limited in quantifying behavior accurately at the individual level but may be valuable for population-level surveillance over time. For population surveillance, particularly in settings with limited resources for conducting health surveys, assessing the accuracy of single or dual-item questions for characterizing behaviors is critical. Opportunities to decrease sitting time in urban areas with high walkability are implicated. Our findings are key for health intervention and policy planning focused on improving sitting-related health outcomes, distinct from interventions promoting other recreational and nonrecreational physical activity, with potential generalizability to other urban, adult populations.

## Figures and Tables

**Table:** 

**1. The activity supported the learning objectives.**				
**Strongly Disagree**				**Strongly Agree**
1	2	3	4	5
**2. The material was organized clearly for learning to occur.**				
**Strongly Disagree**				**Strongly Agree**
1	2	3	4	5
**3. The content learned from this activity will impact my practice.**				
**Strongly Disagree**				**Strongly Agree**
1	2	3	4	5
**4. The activity was presented objectively and free of commercial bias.**				
**Strongly Disagree**				**Strongly Agree**
1	2	3	4	5

## Appendix

Mean Self-Reported Day and Evening Sitting Times, by Demographics, Physical Activity Transit Survey, 2010–2011. This file is available for download as a Microsoft Word document.

## Post-Test Information

To obtain credit, you should first read the journal article. After reading the article, you should be able to answer the following, related, multiple-choice questions. To complete the questions (with a minimum 75% passing score) and earn continuing medical education (CME) credit, please go to http://www.medscape.org/journal/pcd. Credit cannot be obtained for tests completed on paper, although you may use the worksheet below to keep a record of your answers. You must be a registered user on Medscape.org. If you are not registered on Medscape.org, please click on the "Register" link on the right hand side of the website to register. Only one answer is correct for each question. Once you successfully answer all post-test questions you will be able to view and/or print your certificate. For questions regarding the content of this activity, contact the accredited provider, CME@medscape.net. For technical assistance, contact CME@webmd.net. American Medical Association's Physician's Recognition Award (AMA PRA) credits are accepted in the US as evidence of participation in CME activities. For further information on this award, please refer to http://www.ama-assn.org/ama/pub/about-ama/awards/ama-physicians-recognition-award.page. The AMA has determined that physicians not licensed in the US who participate in this CME activity are eligible for ***AMA PRA Category 1 Credits*** ™. Through agreements that the AMA has made with agencies in some countries, AMA PRA credit may be acceptable as evidence of participation in CME activities. If you are not licensed in the US, please complete the questions online, print the AMA PRA CME credit certificate and present it to your national medical association for review.

## Post-Test Questions

### Article Title: Self-Reported Sitting Time in New York City Adults, The Physical Activity and Transit Survey, 2010-2011

**CME Questions**

1. You are seeing a 55-year-old business executive whose main complaint is that "my life is too stressful." He has been working at least 6 days per week and puts in approximately 11 hours per day. His workday consists of meetings and working on his computer, and he estimates that he is sitting for all but 1 hour per day. What can you tell this patient regarding sedentary behavior and health outcomes?
  - Sitting has been linked with cardiovascular mortality
  - Sitting closely shares demographic determinants with recreational physical activity
  - Sitting is associated with increased lipoprotein lipase activity in skeletal muscle
  - There is conclusive evidence that higher levels of sedentary activity promote negative health outcomes, even among adults who exercise frequently
2. What should you consider regarding data on sitting time in the current study as you evaluate this patient?
  - Mean self-reported sitting time was approximately 2 hours per day
  - Mean accelerometer-reported sitting time was approximately 8 hours per day
  - The correlation between self-reported and accelerometer-reported sitting time was strongest at night
  - Age and race/ethnicity were not significant factors in the correlation between self-reported and accelerometer-reported sitting time
3. Which of the following variables was ***most*** significantly associated with a higher mean duration of self-reported sitting time in the current study?
  - Higher educational attainment
  - Female gender
  - Hispanic ethnicity
  - Foreign-born status
4. Which of the following variables was ***most*** significantly associated with a higher mean duration of accelerometer-reported sitting time in the current study?
  - Older age
  - Male gender
  - Non-Hispanic black race
  - Normal weight vs overweight or obese

**Evaluation**
