# Supplementary material for: Self-Reported Sitting Time in New York City Adults, The Physical Activity and Transit Survey, 2010–2011
Source: Prev Chronic Dis. 2015 May 28;12:E85. doi: 10.5888/pcd12.140488 (PMC4454414; doi:10.5888/pcd12.140488)
Supplement: Supplementary file 1 [file 14_0488_01.docx]

| Table S1: Mean Self-Report of Day and Evening Sitting Times, by Demographics, Physical Activity Transit Survey 2010-11 | | | | | | | | | | |
| --- | --- | --- | --- | --- | --- | --- | --- | --- | --- | --- |
|  | Self-Reported Day Sitting^a^ | | | Self-Reported Evening Sitting^b^ | | | Difference b/t Day vs. Evening Sitting (minutes/day) | | | % of Sitting Time that is Day Sitting |
| Demographic Characteristic | Mean | 95% CI | p-value | Mean | 95% CI | p-value | Mean | 95% CI | p-value |  |
| Overall | 243 | (235, 251) | n/a | 180 | (174, 186) | n/a | 63 | (55, 70) | n/a | 57% |
| Age Group |  |  |  |  |  |  |  |  |  |  |
| 18-24 | 226 | (203, 249) | 0.05 | 180 | (164, 195) | 0.90 | 46 | (23, 69) | 0.06 | 56% |
| 25-44 | 253 | (239, 267) | Ref | 181 | (171, 191) | Ref | 72 | (58, 86) | Ref | 58% |
| 45-64 | 244 | (230, 258) | 0.37 | 173 | (165, 182) | 0.26 | 71 | (58, 84) | 0.89 | 59% |
| 65+ | 226 | (210, 241) | **0.01** | 193 | (181, 205) | 0.13 | 33 | (19, 46) | **<.001** | 54% |
| Sex |  |  |  |  |  |  |  |  |  |  |
| Male | 250 | (237, 263) | Ref | 190 | (182, 199) | Ref | 60 | (47, 72) | Ref | 57% |
| Female | 236 | (226, 246) | 0.10 | 171 | (164, 178) | **<.001** | 65 | (56, 75) | 0.50 | 58% |
| Race |  |  |  |  |  |  |  |  |  |  |
| Non-Hispanic White | 280 | (269, 291) | Ref | 185 | (177, 192) | Ref | 95 | (84, 107) | Ref | 60% |
| Non-Hispanic Black | 241 | (225, 257) | **<.001** | 200 | (186, 214) | 0.06 | 41 | (25, 58) | **<.001** | 55% |
| Hispanic | 174 | (160, 188) | **<.001** | 150 | (139, 161) | **<.001** | 24 | (12, 36) | **<.001** | 54% |
| Non-Hispanic Asian | 278 | (249, 307) | 0.91 | 193 | (176, 211) | 0.38 | 85 | (55, 116) | 0.53 | 59% |
| Other | 256 | (204, 309) | 0.39 | 194 | (158, 229) | 0.63 | 63 | (13, 113) | 0.21 | 57% |
| Poverty/Income^a^ |  |  |  |  |  |  |  |  |  |  |
| <200% FPL | 203 | (190, 216) | **<.001** | 172 | (163, 182) | **0.01** | 31 | (19, 42) | **<.001** | 54% |
| 200-399% FPL | 239 | (221, 256) | **<.001** | 181 | (167, 196) | 0.37 | 57 | (40, 74) | **<.001** | 57% |
| 400+% FPL | 303 | (289, 317) | Ref | 189 | (180, 198) | Ref | 114 | (99, 128) | Ref | 62% |
| Education |  |  |  |  |  |  |  |  |  |  |
| Less than high school | 176 | (156, 197) | **<.001** | 151 | (135, 167) | **<.001** | 25 | (11, 39) | **<.001** | 54% |
| Grade 12 or equivalent | 207 | (192, 222) | **<.001** | 176 | (166, 187) | **<.001** | 30 | (14, 47) | 0.08 | 54% |
| Some college | 250 | (236, 264) | **<.001** | 194 | (184, 205) | **<.001** | 55 | (40, 71) | 0.44 | 56% |
| College graduate | 302 | (288, 315) | Ref | 189 | (180, 198) | Ref | 113 | (99, 127) | Ref | 62% |
| Nativity |  |  |  |  |  |  |  |  |  |  |
| U.S. born | 263 | (253, 273) | Ref | 191 | (184, 199) | Ref | 72 | (62, 82) | Ref | 58% |
| Foreign born | 220 | (207, 233) | **<.001** | 168 | (159, 176) | **<.001** | 52 | (40, 64) | **0.02** | 57% |
| Body Mass Index, kg/m^2^ |  |  |  |  |  |  |  |  |  |  |
| Underweight/Normal, <25 | 255 | (241, 269) | Ref | 182 | (173, 191) | Ref | 73 | (60, 87) | Ref | 58% |
| Overweight, 25-<30 | 240 | (227, 253) | 0.12 | 181 | (172, 191) | 0.96 | 59 | (46, 71) | 0.11 | 57% |
| Obese, 30+ | 227 | (213, 241) | **0.01** | 177 | (166, 188) | 0.51 | 50 | (37, 64) | **0.02** | 56% |
| ^a^Day sitting was assessed with the question, “On an average day during the last 7 days, from the time you woke up to around 5 o’clock in the evening, how many hours or minutes did you spend sitting?” | | | | | | | | | | |
| ^b^Evening sitting was assessed with the question (following the daytime sitting question), “And from 5 o’clock in the evening to the time you went to bed on an average day during the last 7 days, how many hours or minutes did you spend sitting?” | | | | | | | | | | |
| Abbreviations: CI – confidence interval, FPL – federal poverty level | | | | | | | | | | |
